# Genotypic diversity and phenotypic traits of *Streptococcus mutans* isolates and their relation to severity of early childhood caries

**DOI:** 10.1186/s12903-017-0406-1

**Published:** 2017-07-14

**Authors:** Remberto Marcelo Argandoña Valdez, Cristiane Duque, Karina Sampaio Caiaffa, Vanessa Rodrigues dos Santos, Maria Luiza de Aguiar Loesch, Natalia Helena Colombo, Rodrigo Alex Arthur, Thais de Cássia Negrini, Marcelo Fabiano Gomes Boriollo, Alberto Carlos Botazzo Delbem

**Affiliations:** 1 Universidad Mayor de San Andrés (UMSA), Foundation Pro-Joven, La Paz, La Paz Bolivia; 2 Department of Pediatric Dentistry and Public Health, São Paulo State University (UNESP), School of Dentistry, Araçatuba, R. José Bonifácio, 1193, Araçatuba, SP CEP: 16015-050 Brazil; 3 Department of Preventive Dentistry, Faculty of Dentistry, Federal University of Rio Grande do Sul, Porto Alegre, RS Brazil; 4 Department of Oral Diagnostic, Area of Microbiology and Immunology, University of Campinas (UNICAMP), School of Dentistry, Piracicaba, SP Brazil; 5 Department of Research and Postgraduate Studies, Area of Pharmacogenetics and Molecular Biology, University of Alfenas (UNIFENAS), Medicine School, Alfenas, MG Brazil

**Keywords:** Early childhood caries, *Streptococcus mutans*, Dental biofilm, Genotypic diversity, Acidogenicity, Aciduricity

## Abstract

**Background:**

Early childhood caries (ECC) is an aggressive condition that can affect teeth of young children. This study aimed to evaluate genotypic diversity and phenotypic traits of *S. mutans* isolated from dental biofilms of children with different caries status in comparison with caries free (CF) children.

**Methods:**

*Streptococcus mutans* strains were isolated from supragingival biofilm samples of CF, ECC and severe-ECC (S-ECC) children and genotyped by arbitrary-primer polymerase chain reaction - AP-PCR. *S. mutans* genotypes were tested for their ability to reduce the suspension pH through glycolysis, to tolerate extreme acid challenge and by their ability to form biofilm. Response variables were analyzed by ANOVA/Tukey or Kruskal-Wallis/Mann-Whitney tests at a 5% of significance.

**Results:**

There was an increase in the prevalence of *Streptococcus mutans* in biofilms with the severity of dental caries. No differences in genotypic diversity and in acidogenicity of genotypes were found among CF, ECC and S-ECC children. *S mutans* strains with genotypes more characteristic for ECC and S-ECC children formed more biofilms than those identified in CF children. The strains isolated from S-ECC children were highly acid tolerant.

**Conclusion:**

Although *S. mutans* genotypic diversity was similar among the groups of children, phenotypic traits of *S. mutans,* especially the acid tolerance response, could explain the severity of early childhood caries.

## Background

Dental caries is a biofilm-associated disease caused by frequent intake of dietary sugars. Fermentation of these sugars by biofilm microbiota leads to acid production, which disrupt microbial homeostasis and cause dissolution of tooth minerals [1]. Mutans streptococci, mainly *Streptococcus mutans,* have been considered as the most important bacteria implicated in dental caries etiology and progression. These microorganisms are frequently isolated from cavitated caries lesions and induce caries formation in sucrose-fed animals [2]. *S. mutans* has important features which contribute to their virulence, such as their ability to metabolize a wide variety of carbohydrates and produce large amounts of acid (acidogenicity), to tolerate extreme acidic environments (aciduricity) and ability to synthesize extracellular polysaccharides (EPS), mainly from sucrose, which improve their adherence to other microorganisms and to tooth surface [3].

Early childhood caries (ECC) is an aggressive condition of dental caries that affects young children. ECC is classified by The American Academy of Pediatric Dentistry (AAPD) as the presence of one or more decayed, missing (due to caries) or filled tooth surfaces in any primary tooth in a child 71 months of age or younger, while severe ECC (S-ECC) is represented by one or more cavitated, missing (due to caries) or filled smooth surface in primary maxillary anterior teeth or decayed, missing or filled surfaces greater than or equal to four (age of 3), five (age of 4) or six (age of 5) [4]. ECC disease has rampant, acute and progressive characteristics and lead to destruction of the primary dentition affecting negatively children’s physical and mental health in addition to increase the risk of new caries lesions in the permanent dentition [5].

It is well known that saliva and dental biofilm harbors different genotypes of *S. mutans* and caries-active individuals seems to have more genotypes than caries-free ones [6–8]. Some studies evaluated genetic diversity of *S. mutans* from ECC children [8–14]. It has been discussed that the simultaneous action of several strains with possibly different cariogenic potential could increase the risk of caries [8]. Phenotypic traits of different *S. mutans* genotypes would be associated with their ability to colonize tooth surface or express factors that could induce the formation of caries lesions [11, 15]. However, it is still unknown if genotypic diversity and phenotypic traits of *S. mutans* are related to different caries status or caries severity in children. Therefore, this study aimed to evaluate genotypic diversity, acidogenicity, aciduricity and biofilm formation of *S. mutans* isolated from dental biofilms of caries free (CF), of ECC and of S-ECC children in an effort to understand caries lesion development at individual level.

## Methods

### Study design

This study was approved by the Research Ethics Committee of Araçatuba Dental School, State University of Sao Paulo, Brazil (CAAE 0041.0.258.000-10). Signed informed consent was obtained from the parents/ legal tutors of children previously to the beginning of the study. Twenty and seven children enrolled in public preschools in peripherical area of the city of Araçatuba (São Paulo, Brazil) participated in the study. Children were included if they were in good general health, without syndromes or chronic systemic diseases. Children whose parents/legal tutors refused to sign the informed consent, or who did not cooperate with the clinical exams, were excluded from the study. Dental examinations were conducted by one examiner (CD) using a mouth mirror and a ball-ended dental probe under a focusable flashlight, after biofilm removal and drying with gauze. World Health Organization (WHO) criteria was used to classify children caries status, considering the total number of decayed, missing or filled teeth surfaces (dmfs). Children were divided into three groups according to their caries status (CF, ECC, and S-ECC) according to AAPD guidelines [4]. All children with dental caries were submitted to restorative/surgical treatment performed by a pediatric dentist, after samples collection.

### Samples collection and microbiological procedures

Supragingival biofilm from CF, ECC and S-ECC children were collected from all buccal and lingual smooth intact surfaces at least 1 h after food intake. Biofilm samples were pooled in order to have a representative sample of each individual. No biofilm was collected from caries cavities in order to avoid contamination with *S. mutans* strains harboring this environment. In order to standardize the amount of biofilm, a sterilized plastic disposable handle (Greiner, Frickenhausen, Germany) with a circular opening of about 1 μL of volume capacity was used for biofilm collection immediately after sample pooling. Biofilm samples were placed in 1 ml of TE buffer (10 mM Tris–HCl, 1 mM EDTA, pH 8.0) that was kept on ice for no longer than 2 h. Biofilm suspensions were vortexed for 1 min, an aliquot of each sample was serially diluted in 0.9% NaCl sterile solution and plated on Mitis Salivarius Agar with 0.2 U Bacitracin (Sigma Aldrich) for the isolation of mutans *streptococci* (MS) [10]. All agar plates were incubated at 37 °C for 48 h in an atmosphere of 5% CO_2_. The number of colony-forming units (CFU) was determined from a representative area of each agar plate yielding 30–300 colonies using a stereoscopic microscope and the results were expressed as CFU/ml. Up to six representative colonies of MS were then selected from agar plates and individually transferred to tubes containing 5 mL of Brain Heart Infusion broth (BHI) which were incubated for 24 h under the same conditions described above. The purity of the cultures was checked by Gram-staining. Aliquots of MS isolated strains were frozen at −20 °C in BHI containing 20% glycerol for further use in molecular analysis [13].

### Genotypic analysis

#### DNA extraction and Polymerase Chain Reaction (PCR)

Frozen aliquots of each MS colony isolated from biofilms were grown on MSB (Mitis Salivarius Agar with bacitracin, as described above) and incubated 37 °C for 24–48 h in an atmosphere of 5% CO_2_. The colonies that grew on BHI agar were incubated into BHI broth (Difco) and incubated at 37 °C for 18 h at the same conditions. Cells from these cultures were then harvested and genomic DNA of MS isolates was extracted using a protocol modified by Nascimento et al. [15]. Briefly, samples were submitted to a lysing solution (extraction buffer and proteinase K) and then purified using chloroform:isoamil-alcohol, followed by DNA precipitation with isopropanol and 70% ethanol. The DNA was resuspended in TE buffer (10 mM Tris–Hcl, 0.1 mM EDTA, pH 7.5, with 10 mg/mL RNAse). DNA was quantified in a spectrophotometer at 260 nm for obtaining a standard concentration of 100 ng of DNA/mL from each isolate. DNA samples were stored at −70 °C for subsequent PCR reactions.

In order to confirm *Streptococcus mutans* molecular identity, DNA of MS isolates were submitted to PCR method, using specific primers for portions of the glucosyltransferase B gene (gtfB) following the bases sequences: 5′ – ACT ACA CTT TCG GGT GGC TTG G – 3′ e 5′ – CAG TAT AAG CGC CAG TTT CAT C – 3′, to amplify a 517 bp DNA fragment. Each PCR mixture contained 5 μl of the DNA template, 5 μl of X 10 PCR amplification buffer (100 mM Tris–HCl, 500 mM KCl, pH 8.3), 0.2 mM of dNTPs (DNA Polymerization Mix), 3.0 mM MgCl_2_, 1 μM of each primer and 2.5 U de Taq *DNA* Polymerase and sterile distilled water to obtain a final volume of 25 μl. Positive and negative controls of PCR reactions were purified genomic DNA of *S. mutans* (ATCC 25175) and sterile water, respectively. The amplification of DNA was performed in a thermocycler (GeneAmp PCR System 2400, Perkin Elmer, Applied Biosystems, USA) with an initial denaturation at 95 °C for 5 min, followed by 30 cycles of denaturation at 95 °C for 30 s, annealing at 59 °C for 30 s and extension at 72 °C for 1 min, besides the final extension at 72 °C for 7 min. The PCR amplification products were separated by electrophoresis in 2% agarose gels in Tris-borate-EDTA (TBE) running buffer (pH 8.0) at 100 V for 45 min. Gels were stained with SYBR Green 1.6% and visualized under ultraviolet light illumination (UltraLum – Labtrade do Brasil). A 100 bp DNA ladder was included as a molecular-size marker in each gel. All PCR reagents were obtained from Invitrogen, Life Technologies, São Paulo, Brazil [16, 17].

#### Arbitrary-Primed Polymerase Chain Reaction (AP-PCR)

Isolates molecularly identified as *Streptococcus mutans* were genotyped by AP-PCR technique. Amplification was performed with primer OPA-02 (5′- TGCCGAGCTG – 3′) [6]. All reactions were processed in a volume of 50 μl, containing 1× PCR buffer, 5 U/μl of Taq DNA polymerase, 10 mM DNTp, 20 μM primer, 50 mM MgCl2 and 2 μl of template DNA [6]. The amplification was performed in the same thermocycler with an initial denaturation at 95 °C for 2 min and 45 cycles consisting of 94 °C for 30 s, 36 °C for 30 s and 72 °C for 1 min, concluding with a final extension of 72 °C for 5 min [16]. Amplicons generated by AP-PCR were analyzed eletrophoretically in 1.5% agarose gel in TBE running buffer and stained with SYBR Gren 1.6%. A 1Kb DNA ladder was used as molecular-size marker. The gels were photographed and their images captured with a digital imaging system (Kodak Digital Science 1D). The molecular weights for each band or amplicon were computed and analyzed by the Sigma Gel software program [16, 17].

### Phenotypic analysis

#### Acidogenicity assays (glycolytic curves)

Frozen stocks of *S. mutans* genotypes (*n* = 14 (CF); *n* = 12 (ECC); *n* = 8 (S-ECC)) identified in the dental biofilms were grown on BHI agar plates and incubated at 37 °C for 48 h in an atmosphere of 5% CO_2_. CFU were collected and inoculated into BHI broth, which was incubated at 37 °C for 18 h. These assays were conducted according protocol described by Arthur et al. [17], to evaluate the ability of *S. mutans* genotypes to lower the suspension pH through glycolysis (pH drop). Aliquots of cultures (approximately 10^8^ CFU/ml) grown in BHI broth were centrifuged and resuspended in 50 mM KCl supplemented with 1 mM MgCl_2_ (Fluka, Steinheim, Germany). The pH of the solution was adjusted to 7.2, and glucose was added to a final concentration of 55.6 mM. The decrease in pH was then assessed during 300 min using a glass electrode previously calibrated with pH standards (pH 4.0 and 7.0). The area under the curve (AUC) for the drop in pH after 300 min considering pH 3.0 as a cut-off point was also determined. The acidogenicity was expressed as AUC (total pH drop) or means/standard deviation of pH for each period of time (0 to 300 min). Analyses were performed in triplicate.

#### Aciduricity assays (acid killing assays)

The ability of *S. mutans* genotypes (*n* = 14 (CF); *n* = 12 (ECC); *n* = 8 (S-ECC)) to tolerate acid challenge was evaluated using the acid killing assay [11, 15, 17, 18]. Briefly, aliquots (approximately 10^8^ CFU/mL) grown for 18 h in BHI broth were transferred into fresh BHI broth and grown to mid-exponential phase (OD550 = 0.5). The suspensions were then centrifuged, and the pellets were washed once with 0.1 M glycine buffer (pH 7.0) (Fluka). In addition, the washed pellets were resuspended in 0.1 M glycine buffer pH 2.8, 5.0 and 7.0 (control). Immediately after resuspension (T0) and after 60 min (T60) of incubation at 37^o^ C, aliquots were serially diluted in phosphate buffer (pH 7.2), plated on BHI agar and incubated at 37^o^ C for 48 h in an atmosphere of 5% CO_2_. Cell viability at each time point was expressed as the percentage of bacterial growth in relation to T0 (100%). Analyses were performed in triplicate.

#### Biofilm assays

Biofilm assays were conducted for *S. mutans* genotypes (*n* = 14 (CF); *n* = 12 (ECC); *n* = 8 (S-ECC)) according to Mattos-Graner et al. [19] with slight modifications. An aliquot from a BHI broth culture of each MS isolate (prepared as described above) was diluted 1:100 in fresh BHI, and 200 μl of this dilution was transferred to sterile polystyrene U-bottom microtiter plates. Plates were incubated for 18 h at 5% CO_2_, and biofilm growth was revealed and quantified by staining with crystal violet. Crystal violet absorbance was determined with a plate reader at 575 nm (Eon Microplate Spectrophotometer, BioTek Instruments, USA). The absorbance (A550) of planktonic cultures grown under the same conditions was measured to monitor growth. Biofilm formation for all strains was measured in triplicate plates.

### Statistical analysis

The studied groups (CF, ECC and S-ECC) were considered as dependent variables. The constant “1” was added to CFU because no growth was detected in some samples. A counts were then transformed to log10 (CFU + 1). Age, dmfs, mutans streptococci counts, acidogenicity and biofilm assays were compared among the groups by ANOVA followed by Tukey tests. Data of cell viability from aciduricity assays were transformed to log_10_ due to data dispersion and expressed as the percentage of bacterial growth (T60) in relation to time zero (T0 = 100%) in pH 2.8, pH 5.0 and pH 7.0. Aciduricity and number of isolates and genotypes of *S. mutans* were compared among the groups by Kruskal-Wallis and Mann-Whitney tests. Electrophoretic bands previously scored in the AP-PCR gels were converted into binary data and submitted to NTSYS-pc software (Applied Biostatistics, Inc.), using coefficient SSM (Simple Matching Coefficient) and UPGMA cluster analysis (Unweighted Pair-Group method with Mathematic Average) to generate dendrogram showing genetic similarity among the bacterial strains isolated from CF, ECC and S-ECC children. Statistical analysis was performed using the program SPSS version 17.1. (*p* < 0.05).

## Results

Twenty-seven children, 12 girls (44.5%) and 15 boys (55.5%) between 36 and 65 months of age (mean 45.21 ± 12.04) were included in this study, corresponding to 10 (37.7%) CF, 9 (33.4%) ECC and 8 (29.6%) S-ECC children. There was no statistical difference among CF, ECC and S-ECC children regarding gender and age. Dmfs values differed statistically among the tested groups with S-ECC children presenting the highest values. Counts of MS in biofilms of ECC and S-ECC children did not differ from each other but they were higher than those found in CF children (Table 1).

**Table 1:** Description of the study population

	CF	ECC	S-ECC	*p* value
Age (months; mean ± SD)	44.4 ± 8.19^A^	45.88 ± 9.42^A^	46.37 ± 6.32^A^	0.67
dmfs* (mean ± SD)	0^A^	3.11 ± 1.83^B^	22.6 ± 23.21^C^	0.00
*S. mutans* isolates n (%)**	30 (78.9) ^a^	32 (91.42) ^a,b^	36 (100) ^b^	0.00
Total *S. mutans* genotypes (identical genotypes)	21(14) ^a^	20(12) ^a^	18(8) ^a^	0.62

*dmsf: tooth surfaces with caries (decay), indicated for extraction (missing) and filled

^A^Different uppercase letters shows statistical difference among CF, ECC and S-ECC groups, according to ANOVA/Tukey tests (*p* < 0.05)

^a^Different lower case letters shows statistical difference among CF, ECC and S-ECC groups, according to Kruskal-Wallis and Mann-Whitney tests (*p* < 0.05)

**Percentage calculated in relation to total number of MS isolated in each tested condition

**Fig. 1:**
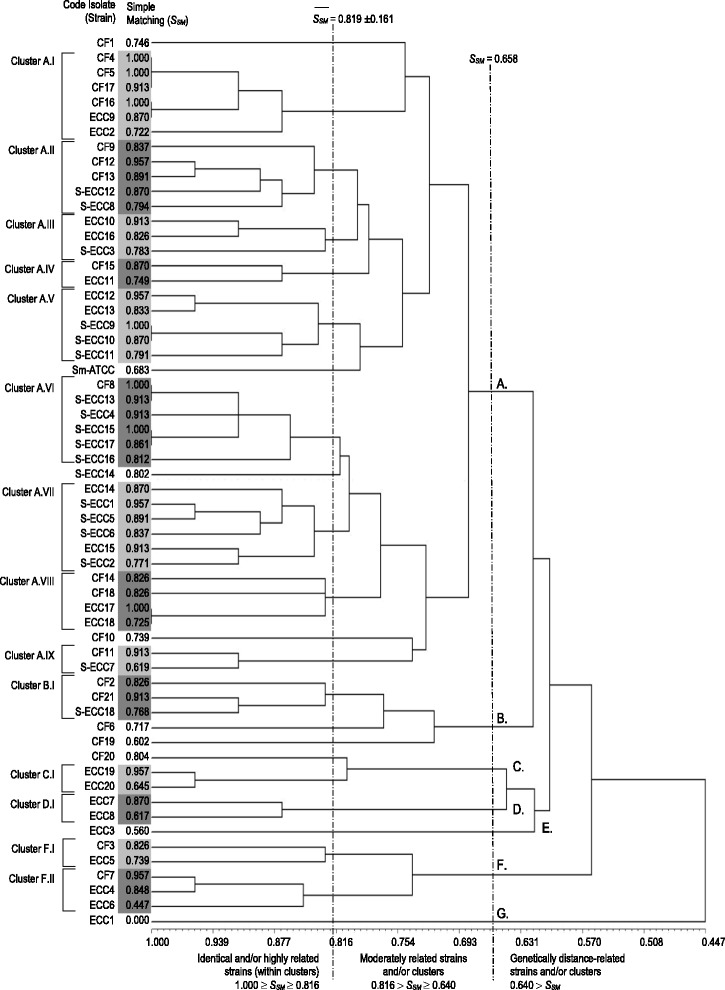
Dendrogram with genetic similarity indices (AP-PCR method, primer OPA-02) verified among *S. mutans* strains sampled from biofilm of CF, ECC and S-ECC children. Individual bands were analyzed by matrices generated by UPGMA analysis using coefficient SSM (simple matching). Tonalities of gray in the dendrogram illustrate identical or highly related isolates (SSM ≥ 0.819 ± 0.161)

One hundred and nine MS strains (38 for CF, 35 for ECC and 36 for S-ECC) were isolated from biofilms. Ninety-eight isolates were molecularly identified as *S. mutans*. S-ECC children presented the highest percentage of *S. mutans* isolates compared with CF and ECC children, which were not different between them (Table 1). Fifty-nine out of 98 *S. mutans* isolates (21 CF, 20 ECC and 18 S-ECC) were re-isolated and genotyped by AP-PCR. A total of 22 clusters (identical or highly related) were detected in biofilm samples after analysis of dendrogram (Fig. 1). Although S-ECC children seems to harbor lesser genotypes compared to other groups, the number of genotypes found in biofilms was not statistically different among CF, ECC and S-ECC children (Table 1). Most of children (53.6%) exhibited two different genotypes. One genotype was found in 35.7% of children, while three or more genotypes were present in 10.7% of children.

**Fig. 2:**
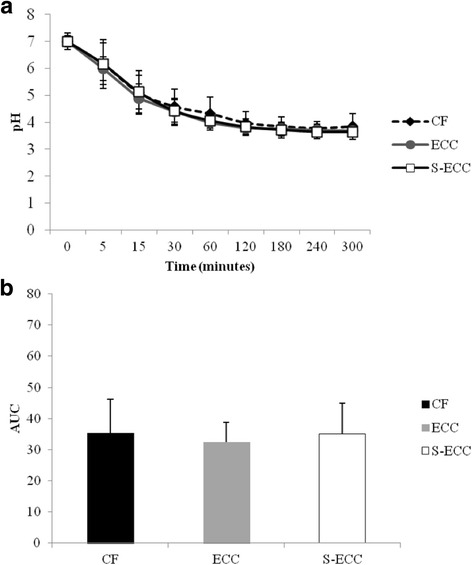
Acidogenicity (acid production). **a** Glycolytic curves. Means of pH values through glycolysis. **b** Means of the area under the curve (AUC) of glycolytic pH fall. *Bars* denote standard deviation for both Figures **a** and **b**. *No difference in pH values among CF, ECC and S-ECC genotypes, for any time point. Different uppercase letters shows statistical difference among CF, ECC and S-ECC genotypes by ANOVA test

**Fig. 3:**
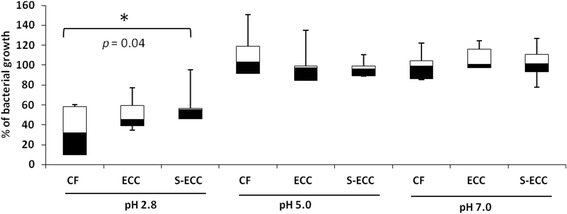
Aciduricity (acid tolerance). Percentage of bacterial growth (T60) in relation to T0 (100%) in pH 2.8, pH. 5.0 and pH 7.0. ^*^ Statistical difference between CF and S-ECC pH by Mann-Whitney tests (*p* < 0.05). *Box plots: bars* indicate minimum and maximum values. *Black* and *white boxes* indicate lower and upper quartiles, respectively. *Line* in the middle of boxes is median

**Fig. 4:**
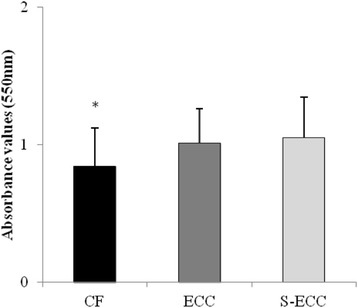
Biofilm formation. Absorbance values (550 nm) obtained for 48 h biofilm of *S. mutans* genotypes from CF, ECC and S-ECC children. Different letters show statistical difference among the groups, according to ANOVA/Tukey tests (*p* < 0.05)

Regarding phenotypic analysis, no statistical difference was observed among the tested groups for acidogenicity of *S. mutans* genotypes considering both pH values at each time point (0 to 300 min) and the values of area under the curve – AUC (Fig. 2; *p* > 0.05). Final pH achieved by genotypes (mean ± SD) of CF children (3.84 ± 0.48) ranged from 4.67 to 3.48; from 4.0 to 3.47 for ECC children (3.69 ± 0.19) and from 3.75 to 3.51 for S-ECC children (3.63 ± 0.12), but no difference was found among the groups evaluated. Considering aciduricity, percentage of growth for *S. mutans* genotypes was not statistically different among CF, ECC and S-ECC children at pH 5.0 and 7.0 (Fig. 3). However, genotypes of S-ECC children presented higher percentage of growth compared with ECC and CF at pH 2.8. Under exposure to pH 2.8 for 60 min, counts of viable cells of *S. mutans* were statistically lower than those found at pH 5.0 and 7.0 for CF, ECC and S-ECC genotypes. Genotypes isolated from ECC and S-ECC children presented higher biofilm formation than those isolated from CF children (Fig. 4).

## Discussion

In the present study, differences were not found in genotypic diversity among CF, ECC and S-ECC children. Therefore, the tested hypothesis 1 was rejected. Available evidences are inconclusive regarding genotypic diversity and caries. Some studies reported a decrease in genotypic diversity in caries-active subjects or absence of difference between caries-free and caries-active children [11] while multiple genotypes were associated with caries activity in infants [7, 9]. In the present study, no difference on *S. mutans* genotypic diversity was found in dental biofilm of caries-active and caries-free children, agreeing with aforementioned investigation [11]. Previous studies have suggested that constant sugar stress provided by dietary sugars, a condition found in caries-active individuals, may select some salivary genotypes make them more prone to colonize dental biofilms [17, 20]. These divergences in genotypic diversities among studies could be attributed to differences in the studied population mainly in respect to their age and caries status. Additionally, altogether, these data reinforce that phenotypic traits may be more important than the presence/absence of specific genotypes in biofilm [11].

In respect to the genotyping of MS isolates, Argimón et al. [21] have suggested that *S. mutans* strains derived from caries-active or caries-free individuals cannot be differentiated only based on the presence or absence of specific genetic elements. In order to circumvent this problem, we chose AP-PCR analysis with primer OPA-02 that showed a considerable number of amplicons and an efficient differentiation of genotypes. Previous studies have proved superior efficiency of OPA-02 in the identification of distinct genotypes when compared with other arbitrary primers [6, 7, 11, 17]. Genetic polymorphism among closely related species is determined by changes in base pairing, by deletion or insertion of new genetic sequences and the cloning transmission from external sources [22]. Some strains of *S. mutans* can acquire various cariogenic properties and antibiotic resistance by transformation [23]. Cvitkovitch [23] suggested that bacterial transformations may occur in environments, which suffer changes and extreme fluctuations in population dynamics, such as the oral cavity. Bacteria in these environments are often exposed to different stress conditions, such as excess or lack of nutrients, low pH, high osmolarity and the use of antimicrobial agents by the host [24]. Therefore, the natural genetic transformation could be considered an important mechanism of cell adaptation to environmental changes, providing microbial resistance, genetic variation and rapid evolution of virulence factors [23, 24]. All these microbial traits could favor survival and proliferation and contribute in the development and/or progression of caries lesions.

A limited number of *S. mutans* genotypes (53.6% with 2 genotypes and 35.7% with one genotype) were observed in this present study. This result is consistent with other reports [13, 14, 25, 26], which found less than 2 genotypes in dental plaque per child. Additionally, in this present study, children had complete deciduous dentition and several studies have demonstrated that *S. mutans* infections are established in the early stages of dentition development and remain stable for several years [25–27].

An important characteristic of virulence of *S. mutans* is its ability to metabolize a variety of dietary carbohydrates such as sucrose and produce a large amount of organic acids by fermentation, which acidify the dental biofilm. In this study, no statistical difference in relation to acidogenicity (pH drop) was found among the *S. mutans* genotypes isolated from dental biofilm of CF, ECC and S-ECC children. Previous studies reported no correlation between caries activity and in vitro acid production of *S. mutans* isolates monitored by records of final pH reached after sugar fermentation [7, 10]. Regarding aciduricity, our data showed that genotypes isolated from S-ECC children are more acid-tolerant under extreme acidic pH challenge (such as the tested pH 2.8 condition) than those isolated from CF and ECC children. This data is in consonance with Lembo et al. [11] that demonstrated that genotypes from caries-active children presented low-susceptibility to acid challenge. However, it is important to mention that no criterion for the assessment of caries lesions severity was adopted in that study [11]. Therefore, we were able to show indeed that acid-tolerance of genotypes is increased with increased caries severity, since no difference was found in cell viability between CF and ECC children under distinct acidic environments. The frequent acidification of oral cavity as a result of sugar fermentation by biofilm microbiota is followed by expression of numerous proteins responsible for maintenance of cell viability by a process called acid-tolerance response – ATR [28]. This mechanism is dependent on a number of virulence traits including the ability to carry out glycolysis at a lower pH [17, 20]. The adaptation process to acidic environments in *S. mutans* is progressive, indicating not a genetic selection but a gradual physiologic process, changing membrane lipid composition, incorporating unsaturated fatty acids into the plasma membrane [29] and affecting activities of membrane ATPases [30]. *S. mutans* controls proton intracellular influx increasing proton extrusion via end-product efflux and F_1_ − F_0_-ATPase activity. The *S. mutans* F_1_ − F_0_-ATPase can operate at low pH much more efficiently than the ATPase of several other competing oral bacterial species such as *Streptococcus salivarius* and *Streptococcus sanguinis* [31]. Besides, *S. mutans* are able to encode several enzymes to protect DNA or repair DNA damage from the harmful effects of intracellular acidification [28]. As a response to all these physiological changes, the frequent pH fall found in oral cavity of S-ECC children allowed a more efficient acid adaptation of their *S. mutans* genotypes, which contributed to the enhanced aciduricity. Considering acidogenicity and aciduricity, it seems based on our results that the latter one plays a more important role on the development of cariogenic biofilms compared with the ability of strains in acid production from dietary sugars.

The present study also evaluated the ability of *S. mutans* genotypes to form in vitro biofilm in a sucrose-rich medium. ECC and S-ECC genotypes formed higher amount of biofilm biomass than CF genotypes. This finding corroborated with those obtained by other researches using animal model [32] and in vitro study [10]. This last study showed that *S. mutans* isolates exhibited variability in their ability to form biofilm, but five of the six high biofilm-forming isolates were obtained from caries-active children [11]. The potential to form biofilm can be also indirectly determined by the production of insoluble extracellular polysaccharides (EPS) [10]. Individuals with high caries activity are often infected by *S. mutans* strains that produce significantly higher amounts of EPS compared to strains infecting caries-free children [10] and adults [7]. EPS improve bacterial adherence to dental surfaces [10]. Additionally, EPS modify the matrix of biofilms making them more porous facilitating the diffusion of acids and carbohydrates throughout the biofilm allowing pH fall in tooth/biofilm interface [33]. It may be possible though that ECC and S-ECC genotypes produce more EPS than CF genotypes. This question will be addressed in further studies. Therefore, hypotheses 2 and 3 could be partially accepted.

In the current study, in agreement with Kouidhi et al. [34], we showed that the detection of *S. mutans* in biofilms increased depending on severity of dental caries, since all MS isolates from S-ECC children were positively identified as *S. mutans*. As discussed above, *S. mutans* are highly acidogenic, aciduric and able to form thick biofilms in the presence of sucrose. Then it is important to discuss that up to 80% of MS isolates were identified as *S. mutans* in biofilm samples of CF children which means that phenotypic traits of *S. mutans* are more important for caries development than their relative numbers in biofilms. This is supported by our data in a way that biofilm forming ability and acid tolerance were higher in genotypes isolated from ECC and S-ECC children.

## Conclusions

Although genotypic diversity was similar in children, regardless of caries status, *S. mutans* genotypes from caries-active children were more acid-tolerant and presented higher ability to form biofilm than those isolated from caries-free children. Acid tolerance seems to be the most important *S. mutans* trait related to the pathogenesis of severe early childhood caries.

## Abbreviations

AP-PCR: Arbitrary-primer polymerase chain reaction
AUC: Area under the curve
CF: Caries free
dmfs: Decayed, missing, filling teeth surface
DNTp: Deoxynucleotide solution mix
ECC: Early childhood caries
EDTA: Ethylenediamine tetraacetic acid
HCl: Cloridric acid
MgCl_2_: Magnesium chloride
NaCl: Sodium chloride
NTSYS: Numerical Taxonomy and Multivariate Analysis System
S-ECC: Severe early childhood caries
SSM: Simple Matching Coefficient
TaqDNA polymerase: Termus aquaticus DNA polymerase
UPGMA: Unweighted Pair-Group Method with mathematic average
